# Multiple Myeloma and the Role of Bisphosphonates in Its Management

**DOI:** 10.7759/cureus.45270

**Published:** 2023-09-14

**Authors:** Nida Tanveer, Sally Hussein, Shravya Pingili, Vijaya Krishna Makkena, Arturo P Jaramillo, Babatope L Awosusi, Javaria Ayyub, Karan Nareshbhai Dabhi, Namra V Gohil, Pousette Hamid

**Affiliations:** 1 Internal Medicine, California Institute of Behavioral Neurosciences & Psychology, Fairfield, USA; 2 Medicine, Kakatiya Medical College, Hyderabad, IND; 3 MBBS, Osmania Medical College, Hyderabad, IND; 4 General Practice, California Institute of Behavioral Neurosciences & Psychology, Fairfield, USA; 5 Pathology and Laboratory Medicine, California Institute of Behavioral Neurosciences & Psychology, Fairfield, USA; 6 Internal Medicine, Medical College Baroda, Vadodara, IND; 7 Neurology, California Institute of Behavioral Neurosciences & Psychology, Fairfield, USA

**Keywords:** bone density, multiple myeloma treatment, bone loss, short term bisphosphonates, lytic bone lesion

## Abstract

An aberrant growth of plasma cells in the bone marrow characterizes the hematological neoplasm known as multiple myeloma, which is typically accompanied by increased bone pain and skeletal-related events such as pathological fractures and/or spinal cord compression. Changes in the bone marrow microenvironment brought on by increased osteoclastic activity and/or decreased osteoblastic activity as a result of myeloma bone disease have a detrimental effect on quality of life. Bone-modifying medications such as bisphosphonates or denosumab are used to treat myeloma bone disease. These substances can lessen bone pain and the chance of pathological fracture, but they do not stimulate the growth of new bone or heal already damaged bone. In order to conduct this study, we searched the PubMed, Google Scholar, and Cochrane databases for complete free papers published in English and studied people over the previous five years, starting in 2018. The search covered randomized clinical trials (RCT), observational studies, meta-analyses, systemic reviews, and conventional reviews. Twenty-five publications are picked after using quality evaluation techniques to determine the type of study. These papers' full-text articles are investigated, examined, and tallied. We spoke about the various treatments for bone damage in multiple myeloma. It was discovered that bisphosphonates lessen the frequency and severity of bone problems. However, we are unsure of their contribution to survival.

Although these medicines enhance life quality, it is unknown if they also increase overall survival. The focus of this study is on several kinds of bone-modifying drugs, their processes of action, the point at which therapy is started, how long it lasts, and any possible mortality advantages.

## Introduction and background

A cancer of the hemopoietic system known as multiple myeloma (MM) is defined by the growth of the plasma cells, which typically occur in the bone marrow but can also be identified in the bloodstream as a solitary plasmacytoma. It is a complex illness that ranges from cells in plasma leukemia to monoclonal gammopathy, which is of uncertain significance [1,2].

Skeletal involvement occurs in more than 85% of MM patients, which can be disastrous [3]. Bone diseases primarily impact the axial skeleton and can have serious skeletal consequences such as compression of the spinal cord and pathologic fractures requiring radiotherapeutic and/or surgical intervention. Bone-modifying medications (bisphosphonates/BPs or denosumab/DENOS) are often used to treat myeloma individuals' usual bone pain and fractures by inhibiting the action of osteoclasts in the bones, but they are not without toxicity. They can be implemented alone or in conjunction with other drugs. Bone-modifying medications such as BPs or DENOS are used to treat myeloma bone disease. These substances can lessen bone pain and the chance of pathological fracture, but they do not stimulate the growth of new bone or heal already damaged bone. Although these medicines enhance life quality, it is unknown if they also increase overall survival. This study focuses on several kinds of bone-modifying drugs, their modes of action, the timing of treatment commencement and maintenance, and any possible longevity advantages [4].

## Review

Methodology

Search for Systematic Reviews

We used MeSH keywords for MM to search the clinical query areas of the PubMed database for relevant articles and systematic reviews. To find literature reviews that the PubMed search could have overlooked, the Cochrane database of reviews was also searched. It has been carried out from the start of the search until the present. PubMed, Google Scholar, Advanced Research, and Snowball Research were also included.

Inclusion/Exclusion Criteria

This overview was open to all systematic evaluations of phase III randomized controlled trials (RCTs) evaluating the impact of therapies on MM. Observational research and other types of study designs were not included. The collected data was from the last five years and was peer-reviewed, and the English language was preferred.

Data Collection

Two impartial reviewers examined all citations and pertinent abstracts before deciding which ones to include in the summary. If the choice to include could not be made from the abstract, a full-text examination was conducted. Consensus was used to settle any conflicts.

A data extraction form was used to extract the data. Each systematic investigation yielded data on patients, intervention, control, and outcomes (PICO). If a meta-analysis was carried out as part of the systematic assessment procedure, quantitative information on outcomes was taken for each PICO question. When many research studies addressed the same PICO issue, we took the most recent and comprehensive thorough review's summary data. We also assessed the process of evaluation's methodological excellence.

Discussion

The most devastating complication of human cancer is metastasis, while the significant affinity for bone results in permanent, severe side effects.

Hypercalcemia renal insufficiency, anemia, and osteolytic bone deterioration, often known as CRAB, are the major clinical symptoms of MM. One of the most frequent side effects of myeloma is myeloma bone disease (MBD), which is referred to as all skeletal-related events (SREs), including bone pain, pathological fractures, and spinal cord compression [5]. Few medicines are currently licensed for the treatment of MM bone disease, which greatly encourages the development of novel therapeutic strategies. Recent studies have highlighted the physiological function of osteocytes in reshaping bones as well as the importance of bone mineral density (BMD) association in the development of MM bone disease and the role of BPs in the progression of the disease. BPs are characterized by their phosphorus-carbon-phosphorus structural backbone and are connected to inorganic pyrophosphates on a molecular level [6]. BPs are synthetic, hydrolysis-resistant compounds with a high affinity for calcium that target regions of high resorption on bone hydroxyapatite surfaces in contrast to inorganic pyrophosphates [7,8]. Generations are another way to categorize BPs, with the first generation consisting of non-nitrogenous medications including clodronate, etidronate, and tiludronate. On the other hand, nitrogen is present in the subsequent generations. Alendronate, neridronate, and pamidronate are examples of medications from the second generation, and risedronate, minodronate, zoledronate, and ibandronate are examples of medicines from the third generation [9].

Following the creation of more potent nitrogen-containing drugs like intravenous pamidronate and zoledronic acid, the MM and BP fields have made significant strides that have improved the patient's quality of life in several areas, including the reduction of bone pain, the prevention of SRE, and hypercalcemia [10,11]. In addition to inducing apoptosis in MM cells, inhibiting the release of bone marrow-derived growth factors like transforming growth factor and insulin-like growth factor into the marrow, downregulating the production of interleukin 6 (IL-6) from the bone marrow stroma, and stimulating T-cell-mediated antiplasma cell activity in the marrow, BPs may also have antimyeloma effects [12]. The mevalonate route of protein prenylation is the mechanism by which BPs limit osteoclast (Oc) activity. IL-1, IL-6, IL-11, IL-3, and IL-17 are produced in the bone as the outcome of interactions between MM cells and T cells. These cytokines stimulate the activity of osteoclasts. The cytokines in question enhance osteoclast activity while reducing osteoblastogenesis, which results in more bone loss. BPs and DENOS both affect receptor activator of nuclear factor kappa-Β ligand (RANKL), which in turn decreases the osteoclast activity [13]. The three most widely used BPs are zoledronic acid, pamidronate, and oral clodronate; however, clodronate is not authorized for usage in the US. Oral BPs have a low absorption rate (1%-5% of the entire dose), but if given on an empty stomach, absorption is greatly increased [14]. BPs have an hour-long half-life, although they may stay in the patient's bones for the rest of their lives. About 70% of ingested BPs are eliminated through the kidneys, and 30% are absorbed by bone. Rapid bone turnover causes an increase in bone uptake [15].

Before each infusion of pamidronate or zoledronic acid, the serum creatinine should be checked [16,17]. Individuals with significant bone disease and pre-existing renal failure (serum creatinine > 3 or estimated CrCl = 30) can receive 90 mg of pamidronate intravenously over 4-6 hours; however, there are no dose recommendations for individuals with pre-existing renal impairment (CrCl = 30-60 ml/min) [18].

Although BPs benefit the patient's standard of life, there is a higher chance that they could contribute to bone erosion of the jaw. This is identified by the mandibular area displaying exposed necrotic bone, which often lasts for eight or more weeks [19]. In contrast to oral-intaking treatment, patients having BPs intravenously had a higher risk of suffering from medication-related osteonecrosis of the jaw (MRONJ) or implant removal [20].

When using BPs, the renal function needs to be watched carefully. Acute tubular necrosis or collapsing focal segmental glomerulosclerosis are the major causes of nephrotoxicity. It is influenced by infusion rate and dosage [21]. Various BPs including pamidronate, zoledronate, alendronate, and ibandronate have been suggested to induce focal segmental glomerular sclerosis (FSGS)[22]. BPs should be stopped if serum creatinine levels increase while under therapy and wait until they reach 10% of the baseline level. Before treatment cessation, patients should be resumed on the same dosage.

While using BPs, the albuminuria level should be monitored every three to six months. A spot urine test for albuminuria should be performed first; if positive, 24-hour urine testing should follow. It is advised to stop taking the medication until renal function has improved if the urine protein level is greater than 500 mg/24 hours. Every three to four weeks, the patient should get a new evaluation [23].

Reduced survival has been linked to impaired renal functions [24,25]. For individuals with renal impairment, DENOS is the primary MBD therapy [26].

BPs cause bone complications due to elevated severe glycation of end products due to bone microdamage from BPs, which results in increased diversity of the matrix of bone and mineral characteristics, and atypical femoral fracture occurs [27-29]. Antiresorptive medication (BPs or DENOS) reduces the risk of SREs but does not promote the production of new bone or the healing of damaged bone. Zoledronic acid every three months is a possibility, but it has to be further investigated in bigger clinical trials. When a disease relapses, we restart the use of bone-modifying drugs. In general, we give a lower dose frequency (i.e., every three months) if the newly diagnosed MM individual reaches better. If tolerated well, bone-modifying drugs can be continued indefinitely in situations of recurrent illness; however, this choice is frequently made case-by-case and following the form of the recurrence and/or the patient's reaction to the initial treatment that was initiated at the time of the relapse [30].

Individual patient features will affect the best treatment option if relapse therapy is necessary [31]. In 280 patients with a lytic bone disease brought on by either MM (n = 108) or breast cancer (n = 172), zoledronic acid (0.4, 2, or 4 mg; 5-min infusion) was compared to pamidronate (90 mg; 2-h infusion) in a randomized phase 2 trial. The main goal was to determine the dosage of zoledronic acid required to cut the proportion of patients who required radiation therapy to 30% or less. Pamidronate and 2 or 4 mg of zoledronic acid both had a comparable percentage of individuals who required radiation therapy (18%-21%). Antiresorptive medication (BPs or DENOS), which reduces the risk of SREs but does not promote the production of new bone or the healing of damaged bone, is the current choice for treating MBD [32]. In a significant phase 3 study, 1648 patients with MM or breast cancer with one lytic lesion received infusions of pamidronate (90 mg) or zoledronic acid (4 mg and 8 mg) every three to four weeks. Over 13 months, the percentage of SREswas the same in each therapy group. In addition to a lower incidence and event rate for radiation treatment to the bone (both generally and in breast cancer patients receiving hormone therapy), the zoledronic acid arm also demonstrated a marginal reduction in skeletal morbidity. Both pamidronate and zoledronic acid were well tolerated. An SRE incidence rate of 1.43 was related to zoledronic acid, compared to a rate of 1.64 for pamidronate, according to a mixed-treatment comparison meta-analysis [33].

Therefore, we advise conducting more studies in the following fields. (1) It should be investigated if bone-anabolic therapy (such as antisclerostin) may be used in conjunction with antiresorptive therapy to treat MBD. (2) For optimal treatment for MBD, bone turnover indicators such as alkaline phosphatase (ALP), osteocalcin, sclerostin, propeptide of type 1 collagen, and P1nP must be confirmed in clinical investigations. (3) High-risk MM patients who are susceptible to active MBD should pursue aggressive and customized targeted treatments. (4) According to the literature, antiresorptive treatment for MBD lasts for two years (or one year if complete response/CR​​​​​​** **or very good partial response/VGPR is obtained); however, it should be investigated if low dosages and reduced frequency (or switching to oral clodronate) may be continued after these periods. (5) For high-risk and aggressive MBD, low-dose BPs and DENOS should be investigated. Zoledronic acid every three months is a possibility, but it has to be further investigated in bigger studies [34-37].

We discovered that one of the areas of general concern was the medical care of bone metastases. Clinical trials and fundamental laboratory research continue to be of utmost importance and demand a deeper and more thorough investigation. This data-driven study demonstrated a significant benefit of a bibliometric evaluation and may help open the door for more bone metastases research. In conclusion, the study of bone tumors appears to have tremendous potential in MM.

Our study has several restrictions. First, we evaluated all included research thoroughly rather than performing a meta-analysis of trials including BPs. Additionally, as only English-language papers were included, we were unable to take into account more research.

Regarding the study's advantages, it is a thorough examination of the research done on the effectiveness of BPs in myeloma patients over the last five years. We also took into account the ongoing studies in the area as well as trials comparing the more recent pharmacological DENOS and its potential efficacy in treating bone deterioration in MM patients. Thus, we provided a systematic summary of both more established research and cutting-edge viewpoints on the application of antiresorptive drugs in the management of bone disease in MM patients.

Therefore, further research is required to better understand the intricate processes taking place in the bone marrow angiogenic niche and to develop coordinated tactics for combating bone marrow angiogenesis across multiple fronts.

## Conclusions

BPs are well-known medications for the medical management of MM and have an excellent risk profile for long-term use. They are useful in lowering bone disease, but it is unclear if they can increase overall survival and progression-free survival. Their usage has drawbacks and restrictions, particularly in individuals with renal impairment. Newer medications, such as DENOS, are becoming more popular. If long-term usage is found to be safe and effective, it may even replace the use of BP in the treatment of MM. The quality of life, mortality, and morbidity are still significantly negatively impacted by MBD.
